# Patterns of Past COVID‐19 and EBV Infection in Primary Headache Disorders: A Population Study

**DOI:** 10.1002/brb3.70858

**Published:** 2025-09-25

**Authors:** Keshet Pardo, Maor Mermelstein, Shlomit Yust‐Kats

**Affiliations:** ^1^ Neurology Department Rabin Medical Center Petach Tikva Israel; ^2^ Faculty of Medical & Health Sciences Tel Aviv University Tel Aviv‐Yafo Israel

## Abstract

**Introduction:**

Viral illnesses are known to trigger chronic headaches. Following the COVID‐19 (coronavirus disease 2019) pandemic, many patients reported prolonged headaches. However, the impact on the incidence of primary headache disorders remains unclear. The aim of our study was to evaluate whether patients with newly diagnosed primary headache disorders had higher rates of prior COVID‐19 and Epstein–Barr virus (EBV) infections, to further explore the impact of viral infections on primary headache disorders.

**Methods:**

A case‐control study using data from the Clalit Health Services database comparing patients with newly diagnosed primary headache disorders, between June 2020 and June 2023, to non‐headache controls, with regard to rates of prior COVID‐19 and EBV infection.

**Results:**

Our cohort comprised 39,693 patients with primary headaches: 30,956 with migraine, 7984 with tension‐type headache (TTH), and 753 with cluster headache (CH). The control group included 69,272 individuals. EBV infection was associated with both migraine (OR 1.13, 95% CI 1.09–1.17) and TTH (OR 1.11, 95% CI 1.03–1.20). However, in a multivariable analysis, only the association with migraine remained significant. COVID‐19 infection was associated with migraine (OR 1.04, 95% CI 1.01–1.07), but lost significance when adjusted for comorbidities. The association between COVID‐19 and TTH was nonsignificant in a univariable analysis (OR 1.05, 95% CI 1.00–1.12). However, in a stratified analysis, COVID‐19 was significantly associated with TTH among patients with anxiety or depression. The CH group showed no significant differences compared to controls.

**Conclusion:**

Different viral infections have varying effects on primary headache disorders: EBV infection is primarily associated with migraine, while COVID‐19 is primarily associated with TTH. These findings underscore the distinct pathophysiological mechanisms underlying these disorders and suggest a differential involvement of the immune system in their development.

## Graphical abstract

This population‐based case‐control study compared 39,693 patients with primary headache disorders to 69,272 controls. We found that past Epstein‐Barr virus (EBV) infection was primarily associated with migraine, while past COVID‐19 infection was mainly associated with tension‐type headache. These findings highlight distinct patterns of viral associations across headache subtypes.

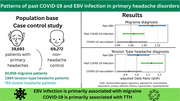

## 1 Introduction

Headache is a common and frequent symptom of COVID‐19 (coronavirus disease 2019) infection (Romero‐Sánchez et al. 2020, Gonzalez‐Martinez et al. 2021). It typically occurs during the acute phase of the disease and is often reported as a presenting symptom (García‐Azorín et al. 2021). The headache associated with COVID‐19 usually exhibits features of either tension‐type headache (TTH) (Porta‐Etessam et al. 2020; Membrilla et al. 2020) or migraine (Caronna et al. 2020). While most COVID‐19 patients experience headaches lasting several days, approximately 20% report persistent headaches over extended periods (Garcia‐Azorin et al. 2022). Headache is also recognized as a component of long‐COVID syndrome (Tana et al. 2022). Most patients with post‐COVID‐19 persistent headache are female and have a prior history of headaches (Sampaio Rocha‐Filho 2022, Carvalho et al. 2024).

The association between viral illnesses and the onset of chronic headache disorders has been documented, particularly in relation to Epstein–Barr virus (EBV) infection, which has been linked to new daily persistent headache (NDPH) in previous studies (Yamani and Olesen 2019, Mack 2004). Chronic headache attributed to systemic viral infections is a recognized syndrome, as defined by the International Classification of Headache Disorders (ICHD‐3) (Arnold 2018), though it is not commonly used in clinical practice.

The question of whether the incidence of primary headache disorders, specifically migraine, increases following COVID‐19 infection remains controversial. Some studies have reported an elevated incidence during the pandemic, while others have found no significant difference (Carvalho et al. 2024, Caronna et al. 2023).

A potential association between primary headache disorders and COVID‐19 infection could provide insights into the pathophysiology of these conditions, enabling better management and reducing the burden of chronic headache disorders. Additionally, if a higher prevalence of primary headache syndromes is observed in individuals following a viral infection, raising awareness about this connection could improve diagnosis and treatment rates for affected individuals.

As of today, a gap remains in our knowledge regarding whether viral infections, such as COVID‐19, increase the risk of primary headache disorders, specifically migraine, TTH, and cluster headache (CH), due to a lack of large population studies.

The goal of our study was to evaluate whether patients with newly diagnosed primary headache disorders had higher rates of prior COVID‐19 infection. To further explore the impact of viral infections on primary headache disorders, we also assessed the rates of prior EBV infection in these patients. To address this question, we conducted a cohort study comparing newly diagnosed primary headache disorders to non‐primary headache controls.

We hypothesize that viral infections may influence the pathophysiology of primary headache disorders and be associated with higher incidence of primary headache disorders.

## 2 Methods

### 2.1 Study Design

We conducted a case‐control study, comparing patients with a new diagnosis of primary headache disorder to controls without any primary headache disorder.

### 2.2 Settings

Our study included patients insured by Clalit Health Services (CHS), the largest of the four health maintenance organizations (HMOs) in Israel. CHS provides primary care and serves both as a healthcare insurer and provider. It manages extensive community care services and operates 14 hospitals, covering both outpatient and inpatient care. CHS provides comprehensive healthcare to approximately 5 million members, representing over half of the country's population. Its electronic database includes continuous real‐time inputs from physicians and healthcare providers, encompassing primary care, community consultation clinics, hospitalizations, laboratory results, imaging studies, and pharmacy records.

### 2.3 Study Population

Our study included all adult CHS members (age > 18) with a new diagnosis of primary headache between June 2020 and June 2023. Primary headache diagnosis was defined based on recorded physician diagnoses as classified by the International Classification of Diseases, Ninth Revision (ICD‐9) codes, and included diagnoses of CH, TTH, and migraine (with or without aura). Additionally, patients who redeemed prescriptions for migraine‐specific medications, such as triptans, gepants, or anti‐CGRP monoclonal antibodies, were also included as part of the migraine subgroup. Exclusion criteria included a diagnosis of a primary headache disorder prior to the study, as defined by either ICD‐9 code or migraine‐specific medications.

The control group consisted of patients matched by age (±5 years) and sex, without any history of primary headache disorder, as defined by either ICD‐9 code or migraine‐specific medications.

### 2.4 Variables

For each patient, we collected data on sex, age, medical history, prior COVID‐19 and EBV infections, COVID‐19 vaccination status, type of headache disorder, prescribed chronic and acute headache medications, and the time between the COVID‐19 infection and primary headache diagnosis.

### 2.5 Data Measurements

We assessed the percentages of past COVID‐19 and EBV infections in both cases and control groups. Past COVID‐19 infection was documented using positive polymerase chain reaction (PCR) test results, while past EBV infection was identified through positive serology tests. Additionally, we measured the percentage of COVID‐19 vaccination among participants. As 97% of the population in Israel received the Pfizer‐BioNTech COVID‐19 vaccine (BNT162b2), we did not differentiate between vaccine types in the analysis. A patient was considered vaccinated if they had received at least two doses of the vaccine.

For the case group, we calculated the time interval between COVID‐19 infection and the diagnosis of the new headache.

For the analysis of comorbidities and risk factors, we compared the entire primary headache group to the control group, as well as each headache subgroup (i.e., migraine, TTH, and CH) with its matched control group.

### 2.6 Data Sources

Data were extracted from the CHS database using Clalit's Data Sharing Platform powered by MDClone (https://www.mdclone.com).

### 2.7 Sample Size and Missing Data

The sample size was based on the available data. There was no missing data in our cohort.

### 2.8 Statistical Analysis

Statistical analysis was conducted using Python (Version 3.9.7, Python Software Foundation, http://www.python.org) within Jupyter Notebook as the integrated development environment. We used the following statistical libraries: Pandas (version: 1.5.2) for data preprocessing and handling data frames, SciPy (version: 1.9.3) for statistical tests such as the chi‐squared test, StatsModels (version: 0.13.2) for logistic regression, NumPy (version: 1.20.3) for numerical operations, and Matplotlib (version: 3.4.3) and Seaborn (version: 0.11.2) for data visualization.

Descriptive statistics were performed to summarize the characteristics of the study population. Categorical variables were presented as frequencies and percentages, while continuous variables were expressed as medians with interquartile ranges (IQR) for non‐normally distributed data or means ± standard deviations (SD) for normally distributed data. Normality was assessed using the Shapiro–Wilk and Kolmogorov–Smirnov tests.

Differences between cases and controls were evaluated using the chi‐square test for categorical variables. Univariable logistic regression analyses were performed to assess the association between each comorbidity, past COVID‐19 or EBV infection, and COVID‐19 vaccination and headache outcomes. Results were reported as odds ratios (ORs) with 95% confidence intervals (CIs).

Variables significantly associated with headache outcomes for migraine and TTH were included in a multivariable logistic regression model to identify independent risk factors. The model was adjusted for comorbidities that showed significant differences between groups in the univariable model, including depression, anxiety, diabetes mellitus, hypertension, hyperlipidemia, and COVID‐19 vaccination status. No variables were forced into the model.

Multicollinearity was assessed using the Variance Inflation Factor (VIF), with values > 5 indicating potential concern. Logistic regression models were evaluated for goodness of fit using the Hosmer–Lemeshow test, and homoscedasticity of residuals was examined using a scatter plot of residuals against predicted probabilities. Results from the multivariable analysis are presented as adjusted ORs (aOR) with 95% CIs and *p* values. A two‐sided *p* value of < 0.05 was considered statistically significant.

### 2.9 Ethical Consideration

The study was approved by the local ethical committee (RMC‐0660‐23). Due to the retrospective noninterventional design of the study, informed consent was not required.

The study followed observational cohort guidelines, according to Strengthening the Reporting of Observational Studies in Epidemiology (STROBE).

## 3 Results

### 3.1 Study Cohort

The initial cohort included 39,745 patients diagnosed with primary headaches between June 2020 and June 2023. We excluded cases of posttraumatic brain injury‐related headache, autonomic cephalalgias other than CH, cyclic vomiting, and NDPH (*n* = 52). This resulted in a final cohort of 39,693 patients included in the analysis.

**FIGURE 1:**
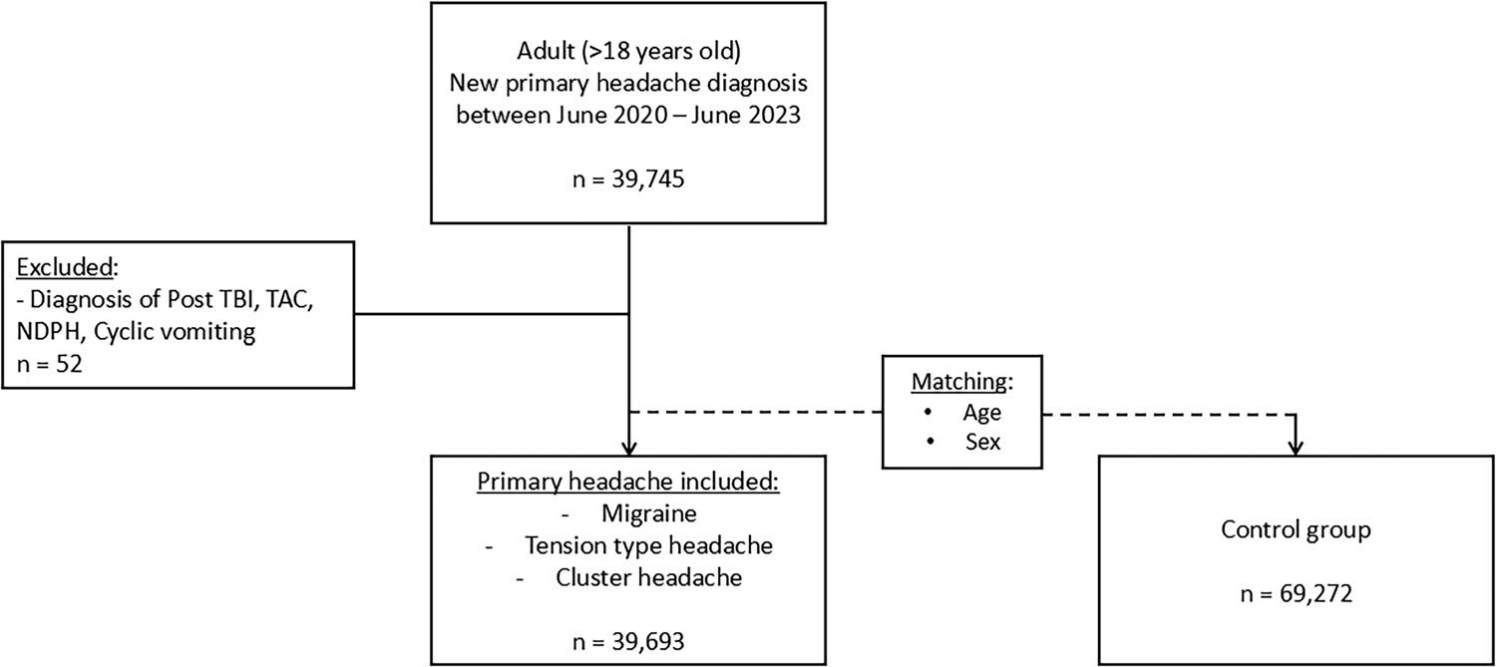
Flowchart. NDPH, new daily persistent headache; TAC, trigeminal autonomic cephalalgia; TBI, traumatic brain injury.

The control group comprised 69,272 individuals matched by age (±5 years) and sex. Figure 1 presents the study flowchart.

### 3.2 Demographic Characteristics

Of the patients included in the final analysis, 30,956 had a diagnosis of migraine, 7984 had TTH, and 753 had CH. The median age at diagnosis was 36.1 years (IQR, 26.9–47.8). The cohort was predominantly female, with 27,914 (70.3%) women. The age and sex distribution of each headache subgroup is detailed in Table 1.

**TABLE 1:** Demographic data of primary headache disorder patients.

	All primary headaches	Migraine	Tension‐type headache	Cluster headache
	*n* = 39,693	*n* = 30,956	*n* = 7984	*n* = 753
Age at diagnosis, years, median (IQR)	36.0 (26.9–47.8)	34.7 (26.2‐45.6)	43.1 (30.7‐58.6)	36.5 (28.0–49.2)
Age at diagnosis, years, mean (SD)	39.1 (15.3)	37.5 (14.2)	45.5 (17.8)	40.2 (15.5)
Sex, Female, *n* (%)	27914 (70.3)	22845 (73.8)	4810 (60.3)	259 (34.4)

Abbreviations: IQR, interquartile range; SD, standard deviation.

The total headache group had higher rates of psychiatric disorders. Depression was present in 5339 (13.5%) of the headache group, compared to 6832 (9.9%) in the control group (*p *< 0.001). Anxiety was present in 10,174 (24.7%) of the headache group compared to 11,990 (17.3%) of the control group (*p *< 0.001). Similar results were observed in each of the headache subgroups, except for the CH group, where only anxiety was significantly more prevalent among CH patients, while depression rates were comparable between the CH group and their matched controls.

Regarding cardiovascular risk, both migraine and TTH patients had higher rates of hypertension and hyperlipidemia compared to the control group. Diabetes mellitus, on the other hand, was less prevalent in both the migraine and TTH groups compared to controls. Obesity was more prevalent in the TTH group compared to the control group and less prevalent in the migraine group compared to controls. No differences were noted for any of the cardiovascular risk factors measured in this study between the CH group and the control group.

All comorbidity data are presented in Table 2.

**TABLE 2:** Comparison of comorbidities, cardiovascular risk factors, and past viral infection and vaccination between the primary headache population and the control.

	Primary headache			Migraine			Tension‐type headache (TTH)			Cluster headache (CH)		
	Primary headache (n = 39,693)	All control (n = 69,272)	*p* value	Migraine (*n* = 30,956)	Non‐migraine controls^a^ (*n* = 54,150)	*p* value	TTH (*n* = 7984)	non‐TTH controls^a^ (*n* = 13,802)	*p* value	CH (*n* = 753)	non‐CH controls^a^ (*n* = 1320)	*p* value
Psychiatric disorders *n* (%)												
Depression	5339 (13.5)	6832 (9.7)	< 0.001	3797 (12.3)	5065 (9.4)	< 0.001	1474 (18.5)	1664 (12.1)	< 0.001	68 (9.1)	103 (7.8)	0.329
Anxiety	10174 (25.6)	11990 (17.3)	< 0.001	7321 (23.7)	9117 (16.8)	< 0.001	2695 (33.8)	2693 (19.5)	< 0.001	158 (21)	180 (13.6)	< 0.001
Cardiovascular risk factors *n* (%)												
Obesity	12233 (30.8)	21797 (31.5)	0.027	8999 (29.1)	16625 (30.7)	< 0.001	3016 (37.8)	4779 (34.6)	< 0.001	218 (29)	393 (29.8)	0.693
Diabetes mellitus	1566 (4.0)	2936 (4.2)	0.019	891 (2.9)	1866 (3.5)	< 0.001	635 (8)	997 (7.2)	0.049	40 (5.3)	73 (5.5)	0.833
Hypertension	6373 (16.1)	9420 (13.6)	< 0.001	3972 (12.8)	6137 (11.3)	< 0.001	2286 (28.6)	3086 (22.4)	< 0.001	115 (15.3)	197 (14.9)	0.831
Hyperlipidemia	9752 (24.6)	15002 (21.7)	< 0.001	6727 (21.7)	10494 (19.4)	< 0.001	2839 (35.6)	4202 (30.4)	< 0.001	186 (24.7)	306 (23.2)	0.434
Past viral infection and vaccination												
Past EBV, *n*, %	6389 (16.1)	10093 (14.6)	< 0.001	5100 (16.5)	8061 (14.9)	< 0.001	1179 (14.8)	1863 (13.5)	0.009	110 (14.6)	169 (12.8)	0.247
Past COVID‐19, *n*, %	15422 (38.9)	26298 (38)	0.004	12184 (39.4)	20860 (38.5)	0.016	2988 (37.4)	4989 (36.2)	0.059	250 (33.2)	449 (34)	0.706
COVID‐19 vaccination, *n*, %	33264 (83.8)	56170 (81.1)	< 0.001	25814 (83.4)	43638 (80.6)	< 0.001	6844 (85.7)	11457 (83)	< 0.001	606 (80.5)	1075 (81.4)	0.591
Time from COVID‐19 to headache diagnosis [days], median (IQR)	316 (157–483)	NA	NA	321 (159–485)	NA	NA	305 (155–477.8)	NA	NA	254 (105–461)	NA	NA

Abbreviations: CH, Cluster headache; COVID‐19, Coronavirus disease 2019; EBV, Epstein–Barr virus; IQR, Interquartile range; NA, not applicable; TTH, Tension‐type headache.

^a^ Control groups were matched by age and sex.

### 3.3 Past EBV Infection

The primary headache group had higher rates of past EBV infection compared to the control group, with 6389 cases (16.1%) in the headache group versus 10,093 cases (14.6%) in the control group (*p *< 0.001). In the subgroup analysis by headache disorder, both the migraine and TTH groups had higher percentages of past EBV infection compared to controls. The OR for EBV infection was 1.13 (95% CI, 1.09–1.17; *p *< 0.001) in the migraine group, and 1.11 (95% CI, 1.03–1.20; *p* = 0.009) in the TTH group. The CH group showed no significant difference from the control group regarding past EBV infection (Tables 2 and 3). In a multivariable analysis (adjusted for comorbidities of depression, anxiety, diabetes mellitus, hypertension, and hyperlipidemia), EBV infection remained independently associated with migraine with an aOR of 1.08 (95% CI, 1.03–1.13; *p* = 0.001), but not with TTH (Table 4 and Supporting Information).

**TABLE 3:** Association between viral infection and vaccination and primary headache disorders.

	OR	95% CI		*p* value
		Lower bound	Upper bound	
Migraine group risk factors				
Past EBV	1.13	1.09	1.17	< 0.001
Past COVID‐19	1.04	1.01	1.07	0.016
COVID‐19 vaccination	1.04	1.00	1.08	0.037
Tension‐type headache group risk factors:				
Past EBV	1.11	1.03	1.20	0.009
Past COVID‐19	1.05	1.00	1.12	0.059
COVID‐19 vaccination	1.23	1.14	1.33	< 0.001
Cluster headache group risk factors:				
Past EBV	1.17	0.90	1.51	0.247
Past COVID‐19	0.96	0.80	1.17	0.706
COVID‐19 vaccination	0.91	0.70	1.18	0.471

*Note*: Univariable analysis.

Abbreviations: CI, confidence interval; COVID‐19, Coronavirus disease 2019; EBV, Epstein–Barr virus; OR, odds ratio.

**TABLE 4:** Association between viral infection and vaccination and migraine and tension‐type headache.

	aOR	95% CI		*p* value
		Lower bound	upper bound	
Migraine risk factors				
Past EBV	1.08	1.03	1.13	**0.001**
Past COVID‐19 infection	1.01	0.97	1.05	0.746
COVID‐19 vaccination	1.00	0.95	1.06	0.959
Tension‐type headache risk factors				
Past EBV	1.01	0.92	1.11	0.840
Past COVID‐19	1.13	1.04	1.23	**0.003**
COVID‐19 vaccination	0.93	0.83	1.04	0.210

*Note*: Multivariable logistic regression, adjusted for comorbidities (depression, anxiety, diabetes mellitus, hypertension, hyperlipidemia).

Abbreviations: aOR, adjusted odds ratio; CI, confidence interval; COVID‐19, Coronavirus disease 2019; EBV, Epstein–Barr virus.

### 3.4 Past COVID‐19 Infection

The primary headache group had higher rates of past COVID‐19 infection compared to the control group, with 15,422 cases (38.9%) in the headache group versus 26,298 cases (38.0%) in the control group (*p* = 0.004). In the subgroup analysis, migraine patients had higher rates of past COVID‐19 infection compared to controls, with an OR of 1.04 (95% CI, 1.01–1.07; *p* = 0.016). For TTH patients, the difference did not reach statistical significance, with an OR of 1.05 (95% CI, 1.00–1.12, *p* = 0.059). The CH group showed no significant difference from the control group regarding past COVID‐19 infection (Tables 2 and 3).

In contrast to past EBV infection, the multivariable analysis (adjusted for comorbidities of depression, anxiety, diabetes, hypertension, and hyperlipidemia) found COVID‐19 to be significantly associated with TTH, with an aOR of 1.13 (95% CI, 1.04–1.23; *p* = 0.003), but not with migraine (Table 4 and Supporting Information).

The time between COVID‐19 infection and headache diagnosis was 316 days (IQR, 157–483) for the entire primary headache group, 321 days (IQR, 159–485) for the migraine group, 305 days (IQR, 155–477.8) for the TTH group, and 254 days (IQR, 105–461) for the CH group (Table 2).

### 3.5 Psychiatric Comorbidities Effect on the Association Between Primary Headache and COVID‐19

We explored the effect of psychiatric comorbidities, specifically anxiety and depression, on the relationship between migraine and TTH diagnosis and COVID‐19 infection through a stratified analysis. In patients with either anxiety or depression, the association between TTH and COVID‐19 was significant, with an OR of 1.12 (95% CI, 1.01–1.24; *p* = 0.032). For patients without these comorbidities, the association was not significant (OR 1.04; 95% CI, 0.97–1.11).

In contrast, for migraine cases, for patients without psychiatric comorbidities, the association between migraine and COVID‐19 was significant, with an OR of 1.04 (95% CI, 1.01–1.08; *p* = 0.018). And for patients with anxiety or depression, the association was not significant (OR 0.99; 95% CI, 0.94–1.05).

### 3.6 COVID‐19 Vaccination

The primary headache group had higher rates of COVID‐19 vaccination, with 33,264 (83.8%) vaccinated patients compared to 56,170 (81.1%) in the control group (*p *< 0.001). Similarly, both the migraine and TTH groups exhibited higher vaccination rates compared to their respective controls. The migraine group had an OR of 1.04 (95% CI, 1.00–1.08; *p* = 0.037), and the TTH group had an OR of 1.23 (95% CI, 1.14–1.33; *p* < 0.001). (Tables 2 and 3). However, the multivariable analysis, adjusted for comorbidities of depression, anxiety, diabetes mellitus, hypertension, and hyperlipidemia, did not reveal a significant association between COVID‐19 vaccination and the new diagnosis of either migraine or TTH (Table 4).

No difference was observed within the CH group compared to the control group (Table 2).

## 4 Discussion

Our study compared patients with newly diagnosed primary headache disorders to non‐headache patients in relation to past viral infections with COVID‐19 and EBV. We found that primary headache disorders were differently influenced by these viral infections. Specifically, EBV was primarily associated with migraine, while COVID‐19 infection showed a stronger association with TTH and a weaker, non‐universal association with migraine.

The COVID‐19 pandemic was speculated to influence various neurological outcomes, including migraine (Xu et al. 2022). Studies evaluating the effect of COVID‐19 on known migraine have yielded inconclusive results, with some reporting worsening of migraines during and after the pandemic (Gentile et al. 2021) or a change in migraine characteristics (Waliszewska‐Prosół and Budrewicz 2021), while others showing negligible effects, such as in a questionnaire‐based study that found no significant worsening of migraine after COVID‐19 infection or vaccination (Melgarejo et al. 2023). However, the focus of our study was not on the effect of COVID‐19 on existing migraine patients but on whether it increases the risk of a new diagnosis of migraine and other headache disorders. While the clinical similarity between headaches attributed to COVID‐19 and migraine suggests a pathophysiological link between the two (Caronna et al. 2020, Bolay et al. 2020), and some reports have described a higher incidence of migraine following COVID‐19 (Xu et al. 2022), our study only found a relatively weak association. The loss of significance in the multivariable analysis and stratified results suggest that COVID‐19 might not be a universal risk factor but may be relevant only under specific conditions, such as the absence of psychiatric conditions. Our results align with recent studies, including a prospective study on several neurological syndromes post‐hospitalization for COVID‐19, which demonstrated a lower incidence of migraine after COVID‐19 compared to influenza (de Havenon et al. 2024), and a retrospective study on COVID‐19 and migraine‐related traits that failed to show any association between the two (Jiang et al. 2024).

In contrast to migraine, COVID‐19 infection was strongly associated with TTH. The stratified analysis showed that this association was more pronounced in patients with psychiatric comorbidities of anxiety and depression, suggesting that these comorbidities may enhance the effect of COVID‐19. TTH is known to be closely associated with both depression and anxiety (Song et al. 2016), and these factors likely contribute to the onset and severity of TTH (Fuensalida‐Novo et al. 2017, Janke et al. 2004). COVID‐19 has also been linked to higher rates of anxiety and depression (Santabárbara et al. 2021, Mazza et al. 2020), possibly due to the inflammatory response and structural changes caused by the virus (Rua et al. 2024), as well as the social isolation related to the pandemic. It is likely that individuals prone to these psychiatric disorders are more susceptible to developing TTH following COVID‐19 infection. This finding could help further our understanding of the pathophysiology of TTH, a disorder that, despite its high prevalence, remains relatively poorly understood (Steel et al. 2021).

Regarding COVID‐19 vaccination, while it initially appeared to be associated with both migraine and TTH, once adjusted for comorbidities, the effect was not statistically significant. There has been an ongoing debate about the neurological adverse effects of COVID‐19 vaccines, including Guillain–Barré syndrome, Bell's palsy, transverse myelitis, and even cerebral venous sinus thrombosis (Hosseini and Askari 2023). Post‐vaccination headache is a common neurologic complaint attributed to COVID‐19 vaccination and has been reported in up to a third of cases, usually in the week following the injection (Castaldo et al. 2022). As for primary headache disorders, some studies have reported worsened chronic headaches (Silvestro et al. 2021) and more severe and prolonged post‐vaccination headaches in the primary headache population (Göbel et al. 2021). However, our results do not support an increased risk of new primary headache diagnoses following COVID‐19 vaccination.

EBV latent infection has been associated with various neurological syndromes, including multiple sclerosis, primary CNS lymphoma (Soldan and Lieberman 2020), and NDPH (Diaz‐Mitoma et al. 1987, Vanast et al. 1987). Several mechanisms have been proposed linking EBV to these conditions, most notably dysregulated immune responses involving both T‐cells and B‐cells (Niller et al. 2008). Our study is the first large‐scale analysis to demonstrate a higher prevalence of past EBV infection in migraine patients, suggesting a potential association between the two. This association could be due to changes in immune responses in migraine patients (Pavelek et al. 2020), and it is possible that more direct pathways linking EBV infection to migraine may be identified in future research.

As for CH, its pathophysiology is thought to involve abnormal activity in the hypothalamus, the trigeminovascular system, and the autonomic nervous system (Hoffmann and May 2018). Current theories do not associate viral infections or immune responses with CH pathophysiology or as triggers for attacks (Rozen and Fishman 2012). Our study corroborates this, showing no significant effect of either COVID‐19 or EBV infection on CH diagnoses. The lack of effect of COVID‐19 infection on CH has also been noted in studies concerning COVID‐19 vaccination, which showed similar headache characteristics in both CH patients and non‐primary headache populations (Göbel et al. 2021).

In this study, we aimed to evaluate the effect of past viral exposure to both COVID‐19 and EBV on primary headache disorders, with the hypothesis that both viruses may modify immune responses and potentially affect the central nervous system (CNS). The pathophysiology of post‐COVID‐19 headache has been suggested to be related to ongoing immune activation with elevated inflammatory cytokines, such as interleukin 6, tumor necrosis factor, and interferon alpha (Straburzyński et al. 2023), while studies regarding EBV have pointed towards direct viral invasion, which leads to chronic inflammation, as a possible mechanism (Jakhmola and Jha 2021); however, further research on the direct or indirect link with primary headache disorders such as migraine and TTH is still needed. Our findings highlight the differing effects of these viruses and their associations with various headache syndromes, underlining not only the differences between the two viruses but also the distinct pathophysiology of these primary headache syndromes. Additionally, this possible association should raise the awareness of clinicians and improve the diagnosis and treatment of these headache disorders.

Our study has several strengths and limitations. The use of the CHS database allowed us to conduct a large‐scale study with a wide population representation. However, the validity of our findings depends on the accuracy of headache diagnoses, which may have been provided by both neurologists and general practitioners (GPs). The positive predictive value of headache diagnoses, particularly migraine, by GPs is high (Tepper et al. 2004), lending reliability to our results. However, the use of large databases could still lead to misclassification due to reliance on ICD codes and prescription data, which might dilute or falsely draw attention to associations of primary headache and viral infections. Additionally, it is possible that other relevant headache syndromes may have been unintentionally included or excluded, potentially affecting the generalizability of the findings. Another limitation of using the CHS database is the use of electronic medical records (EMR) that fail to capture headache activity or severity or symptoms during COVID‐19 infection. The comparison between COVID‐19 and EBV in our study also presents a limitation, primarily due to differences between the two viruses and their documentation, in particular, the timing of acute EBV infection, which remains unknown. However, despite this limitation, we hoped that the comparison, albeit partial, would still provide valuable insights into past viral infections. Finally, the results of our study demonstrate a significant, albeit small effect. With the use of a large data sample, there might be a risk of detecting statistically significant differences that may not be clinically meaningful. We believe that since we are describing disorders that are highly prevalent in the population, even the relatively small effect is noteworthy.

## 5 Conclusion

Different viral infections appear to have varying effects on primary headache disorders. Our study suggests an association between EBV infection and migraine and an association between COVID‐19 and TTH, particularly among patients with psychiatric comorbidities such as anxiety and depression. No significant effect was observed for CH in relation to past viral infections. These findings underscore the distinct pathophysiological mechanisms underlying these headache disorders and suggest differential involvement of the immune system in their development.

## Author Contributions

**Keshet Pardo**: conceptualization, data curation, formal analysis, writing – original draft. **Maor Mermelstein**: conceptualization, writing – review and editing. **Shlomit Yust‐Kats**: conceptualization, writing – review and editing.

## Conflicts of Interest

The authors declared no conflicts of interest.

## Peer Review

The peer review history for this article is available at https://publons.com/publon/10.1002/brb3.70858

## Supporting information




**Supplementary Material**: brb370858‐sup‐0001‐SuppMat.docx

**Supplementary Material**: brb370858‐sup‐0002‐Checklist.docx

## Data Availability

The data that support the findings of this study are available from the corresponding author upon reasonable request.
