# Supplementary material for: Patterns of Past COVID‐19 and EBV Infection in Primary Headache Disorders: A Population Study
Source: Brain Behav. 2025 Sep 25;15(9):e70858. doi: 10.1002/brb3.70858 (PMC12463712; doi:10.1002/brb3.70858)

**Supplements - Patterns of past COVID-19 and EBV infection in primary headache disorders: A population study**

^1,2^Keshet Pardo, ^1,2^Maor Mermelstein, ^1,2^Shlomit Yust-Kats

Neurology department, Rabin Medical center, Petach Tikva, Israel

Faculty of medical & health sciences, Tel Aviv University, Israel

Corresponding author: Dr. Keshet Pardo, [keshetpardo@gmail.com](mailto:keshetpardo@gmail.com), +972-543013307,

Address: Zeev Jabotinsky St 39, Petah Tikva, 4941492

ORCID ID: 0000-0001-5056-2368

| **Table 1S** Multivariable analysis for risk of migraine | | | | |
| --- | --- | --- | --- | --- |
|  | aOR | 95% CI | | P value |
|  |  | Lower bound | upper bound |  |
| Past COVID-19 infection | 1.01 | 0.97 | 1.05 | 0.746 |
| COVID-19 vaccination | 1.00 | 0.95 | 1.06 | 0.959 |
| Past EBV infection | 1.08 | 1.03 | 1.13 | 0.001 |
| Depression | 1.13 | 1.06 | 1.19 | <0.001 |
| Anxiety | 1.49 | 1.42 | 1.56 | <0.001 |
| Obesity | 0.88 | 0.85 | 0.91 | <0.001 |
| Diabetes mellitus | 0.73 | 0.65 | 0.81 | <0.001 |
| Hypertension | 1.22 | 1.16 | 1.30 | <0.001 |
| Hyperlipidemia | 1.15 | 1.10 | 1.21 | <0.001 |
| Multivariable logistic regression, no variable was forced into the model  aOR, adjusted odds ratio; CI, confident interval | | | | |

| **Table 2S** Multivariable analysis for risk of tension-type headache | | | | |
| --- | --- | --- | --- | --- |
|  | aOR | 95% CI | | P value |
|  |  | Lower bound | Upper bound |  |
| Past COVID-19 | 1.13 | 1.04 | 1.23 | 0.003 |
| COVID-19 vaccination | 0.93 | 0.83 | 1.04 | 0.210 |
| EBV | 1.01 | 0.92 | 1.11 | 0.840 |
| Depression | 1.19 | 1.08 | 1.32 | 0.001 |
| Anxiety | 2.02 | 1.86 | 2.20 | <0.001 |
| Obesity | 1.00 | 0.93 | 1.08 | 0.916 |
| Diabetes mellitus | 0.89 | 0.77 | 1.02 | 0.097 |
| Hypertension | 1.59 | 1.44 | 1.76 | <0.001 |
| Hyperlipidemia | 1.16 | 1.06 | 1.26 | 0.001 |
| Multivariable logistic regression, no variable was forced into the model  aOR, adjusted odds ratio; CI, confident interval | | | | |

**Hosmer-Lemeshow goodness-of-fit test:**

**Multivariable analysis for risk of migraine:** P-value: 0.897

**Multivariable analysis for risk of tension-type headache:** P-value: 0.991

**Homoscedasticity of Residuals:**

**Multivariable analysis for risk of migraine:**


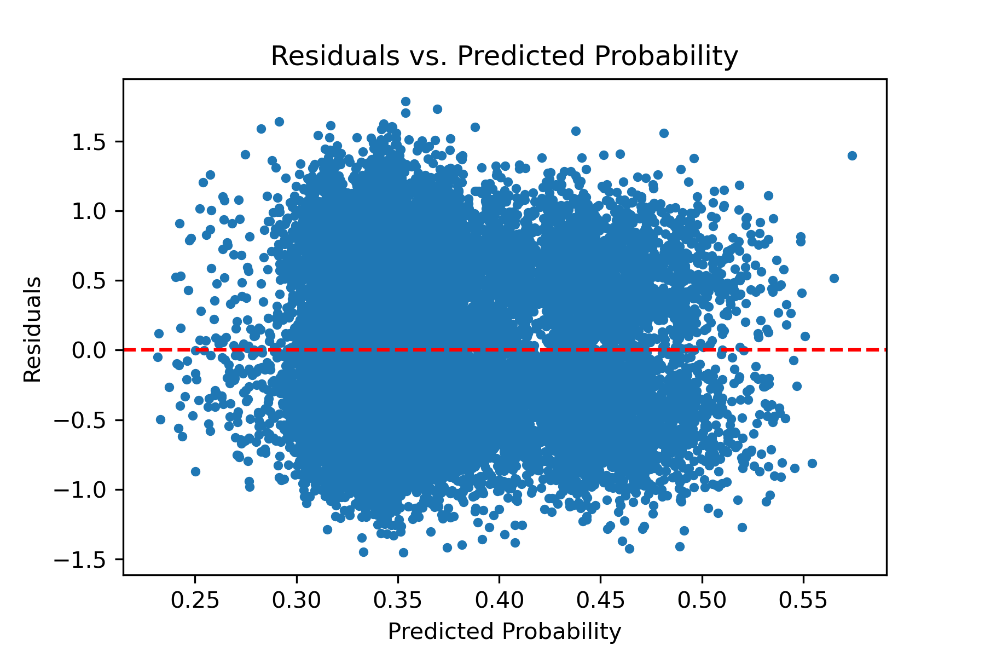


**Multivariable analysis for risk of tension-type headache:**


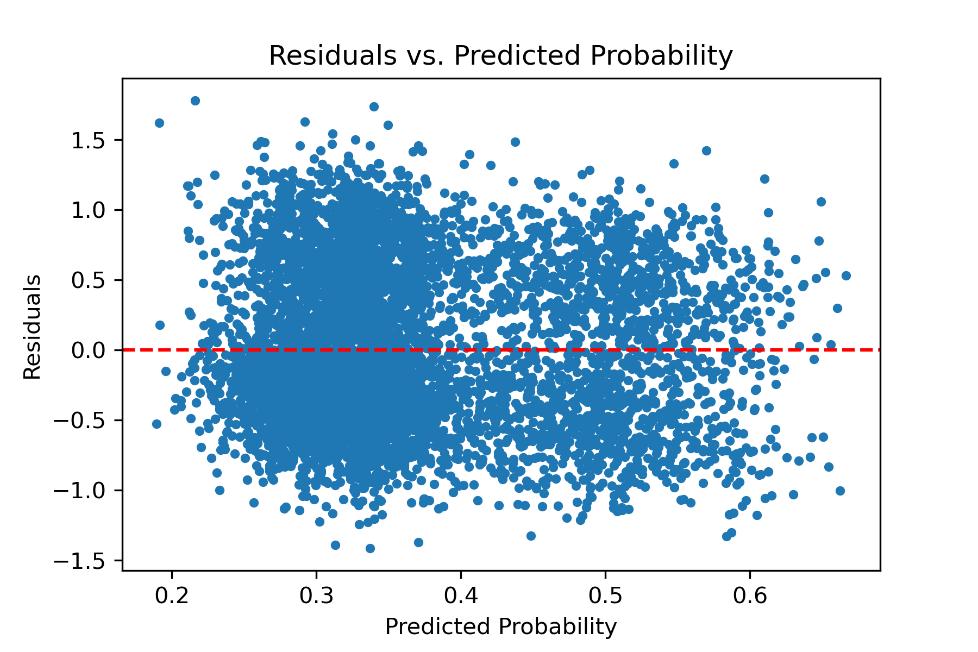

Supplement: Supplementary file 1 — Supplementary Material: brb370858‐sup‐0001‐SuppMat.docx [file BRB3-15-e70858-s001.docx]
